# Medication Complexity and Insomnia Severity in Psychiatric Outpatients: A Moderation Analysis Across Mood and Non-Mood Disorders

**DOI:** 10.3390/clinpract16100189

**Published:** 2026-10-12

**Authors:** Vassilis Martiadis, Enrico Pessina, Azzurra Martini, Marco Marzolla, Chiara Bergesio, Francesca Barbaro, Alex Cavallo, Fabiola Raffone, Carlo Ignazio Cattaneo

**Affiliations:** 1 Department of Mental Health, ASL Napoli 1 Centro, 80125 Naples, Italy; 2 Department of Mental Health, ASL Cuneo 2, 12042 Bra, Italy; 3 Casa di Cura San Michele, 12042 Bra, Italy; 4 Department of Mental Health, ASL Biella, 13900 Biella, Italy; 5 Department of Psychiatry, University of Campania “L. Vanvitelli”, 80145 Naples, Italy

**Keywords:** insomnia, Insomnia Severity Index, psychotropic medication, mood disorders, moderation, community mental health

## Abstract

**Background:** Insomnia is common and occurs across psychiatric diagnoses. A prior analysis of this outpatient cohort found that psychotropic medication complexity was associated with insomnia severity, but whether this association is stronger in particular diagnostic groups was not examined. We preregistered a moderation analysis to test whether mood disorder status modifies the association between medication complexity and insomnia, and additionally examined modification by sex and by insomnia domain. **Methods:** A secondary analysis was performed of cross-sectional, routinely collected data from 502 adult outpatients (from an initial cohort of 506) at an Italian community mental health centre. The primary model regressed the Insomnia Severity Index (ISI) total score on medication complexity × mood disorder status (depressive and bipolar vs. other psychiatric disorders), adjusting for age, sex and education; it was preregistered on the Open Science Framework before any interaction model was estimated. **Results:** Medication complexity was associated with the ISI total (b = 1.54 per class, 95% CI 0.92 to 2.16; attenuated but retained after benzodiazepine adjustment, b = 1.00, *p* = 0.009). The prespecified medication complexity × mood interaction was not statistically significant (b = −1.02, 95% CI −2.25 to 0.21, *p* = 0.103). The medication complexity × sex interaction was also non-significant (b = 0.28, *p* = 0.64), and the moderation did not differ between ISI domains (three-way interaction *p* = 0.67). Benzodiazepine prescription was independently associated with the ISI (b = 1.87, *p* = 0.011). **Conclusions:** The prespecified hypothesis of a stronger medication complexity–insomnia association in mood disorders was not supported. Because no equivalence margin was prespecified, the analysis cannot establish that the association is equivalent across groups; it indicates only that the hypothesised stronger association was not detected. Findings are exploratory with respect to mechanism and should be read in light of the cross-sectional design and probable confounding by indication.

## 1. Introduction

Insomnia is among the most common and disabling complaints in psychiatric outpatient care, affecting a large proportion of patients across diagnostic boundaries. It is often conceptualised through transdiagnostic models, in which disturbed sleep is an active, self-reinforcing process that maintains and amplifies psychopathology rather than being a passive symptom of the primary disorder [1,2]. Consistent with this view, persistent insomnia predicts the onset, recurrence and persistence of mood and other common mental health conditions, and its relationship with psychiatric illness appears bidirectional [3,4].

In routine community psychiatric care, patients frequently receive several concurrent psychotropic medications, and the number of distinct medication classes (here termed “medication complexity”) has been proposed as a pragmatic marker of overall treatment burden. Such regimens frequently combine agents prescribed for core symptoms, for sleep and for agitation, so the number of concurrent classes reflects the overall prescribing context rather than the effect of any single drug. In a consecutive cohort assessed at an Italian community mental health centre, one of the community services established by the 1978 Italian mental health reform, which continue to operate under significant organisational and resource constraints [5], clinically significant insomnia was present in approximately 40% of outpatients, and medication complexity was independently associated with clinically significant insomnia, an association that was attenuated after adjustment for benzodiazepine prescribing [6].

The index study [6] reported only main effects for the sample as a whole and did not examine whether the association between medication complexity and insomnia is stronger in particular diagnostic groups. This distinction is clinically relevant. Prescribing complexity may track illness severity more closely in mood disorders, in which sleep disturbance is also more tightly coupled to the primary illness and predicts its course [3,4]; if so, the association would be expected to be stronger in this group. The competing prediction is that the association is broadly similar across diagnoses. Distinguishing these possibilities has implications for whether insomnia assessment should be prioritised by diagnosis or applied across the caseload.

We therefore preregistered a moderation (effect modification) analysis of the same cohort, with an analytic aim distinct from that of the index study. We hypothesised that the association between medication complexity and insomnia would be stronger in patients with mood disorders (H1). We further tested whether sex modifies this association (H2) and whether any moderation acts preferentially on the impact/distress rather than the nocturnal component of insomnia (H3).

## 2. Materials and Methods

### 2.1. Design, Data Source, and Registration

This is a preregistered secondary analysis of anonymised, routinely collected clinical data from adult outpatients (aged 18 years and over) who attended the Community Mental Health Centre in Bra (Department of Mental Health, ASL Cuneo 2, Italy) between 1 September and 30 November 2025, with one observation per consecutively assessed patient and no further selection. This dataset was previously analysed for the index study [6], which reported the descriptive statistics and main-effect models of the cohort; the present moderation analyses were neither conducted nor reported there, and the shared provenance of the data is stated explicitly. Reporting follows the STROBE guidance for observational studies. The analysis plan was preregistered on the Open Science Framework before any interaction model was estimated (registration: https://osf.io/z6vu8; embargoed until 31 July 2026; DOI 10.17605/OSF.IO/Z6VU8 active on public release). The eating-disorder group (n = 4) was excluded from all models, as prespecified, because the group was too small to contribute informatively to the models and to keep the non-mood comparison group clinically coherent; this yielded an analytic sample of 502. Data were exported from the clinical information system using a visit-date query and de-identified before analysis by removing direct identifiers and date fields; data-quality checks included range checks for the ISI items and confirmation of a single record per patient. There were no missing data on the study variables.

### 2.2. Variables

The primary outcome was the total score on the Insomnia Severity Index (ISI; range 0 to 28, analysed as a continuous variable) [7,8], administered in its validated Italian version [9]. The ISI is a seven-item self-report scale referring to the previous two weeks, with each item rated from 0 to 4. Primary diagnoses were established by psychiatrists using the Structured Clinical Interview for DSM-5 (SCID-5) [10]. The two prespecified secondary outcomes were the nocturnal domain (items 1a to 1c) and the impact/distress domain (items 2 to 5). The focal predictor was medication complexity: the number of distinct psychotropic medication classes prescribed (range 0 to 5), among antidepressants, antipsychotics, antiepileptics or mood stabilisers, lithium, benzodiazepines, Z-drugs and other psychotropics. The measure was based on prescription records and did not capture dose, timing, indication, duration or actual use, and, because benzodiazepines are among the counted classes, the benzodiazepine-adjusted analyses only partly decouple the two. The primary moderator was mood disorder status (mood = depressive and bipolar disorders; non-mood = anxiety, adjustment, psychotic, obsessive–compulsive and personality disorders, and attention-deficit/hyperactivity disorder); the secondary moderator was sex. Covariates were age, sex and years of education, and benzodiazepine prescription (yes/no) was a prespecified sensitivity covariate.

### 2.3. Statistical Analysis

The primary model was a linear regression of the total ISI score on the medication complexity × mood interaction, adjusted for age, sex and education, with the continuous predictors centred on the grand mean; the confirmatory target was the interaction term (coefficient, 95% confidence interval [CI], incremental change in R^2^ and two-sided *p* value). We also estimated the medication complexity slope within each mood group (simple slopes) and the medication complexity main effect with and without adjustment for benzodiazepine prescription. To test H2 as formulated, we fitted the medication complexity × sex interaction. To test H3, rather than fitting the two domains separately, we fitted a single joint model on the standardised domain scores stacked within patients (linear mixed model with a random intercept per patient) and tested the three-way medication complexity × mood × domain term. Prespecified sensitivity analyses added benzodiazepine prescription and psychiatric comorbidity, used heteroscedasticity-consistent (HC3) standard errors, and refitted the model as an ordinal logistic regression on the ISI severity categories, with the proportional-odds assumption assessed by a Brant test. The secondary family (the medication complexity × sex interaction and the three-way medication complexity × mood × domain interaction) was controlled with the Benjamini–Hochberg false discovery rate; the single confirmatory test, the medication complexity × mood interaction, was not corrected. The medication complexity × mood interaction (H1) was the preregistered confirmatory test; the medication complexity × sex interaction (H2) and the joint domain model (H3) were specified during revision, in response to peer review, to test these hypotheses more directly, and are therefore reported as non-preregistered analyses. As exploratory analyses, we estimated the medication complexity slope within each individual diagnosis. Linearity, residual assumptions and influence (Cook’s distance) were inspected. Because the sample size was fixed, we report sensitivity rather than an a priori calculation: with *N* = 502, the analysis had approximately 80% power to detect an interaction of f^2^ ≈ 0.016 (a small effect by Cohen’s benchmarks) or larger; the observed effect (f^2^ = 0.005) was below this threshold, so a very small true interaction cannot be excluded. A non-significant interaction was therefore interpreted as failure to detect the hypothesised effect, not as evidence of equivalence, for which no margin was prespecified. Analyses were performed using IBM SPSS Statistics, version 22 (IBM Corp., Armonk, NY, USA).

## 3. Results

### 3.1. Sample

Characteristics of the analytic sample (*N* = 502; 245 patients with mood disorders and 257 with non-mood disorders) are summarised in Table 1; full descriptive statistics for the cohort are reported in the index study [6]. The ISI total was higher, on average, in patients with mood disorders (13.3 ± 7.0) than in those with non-mood disorders (11.2 ± 6.9) (See Supplementary Material).

### 3.2. Medication Complexity and the Isi Total

Medication complexity was associated with the ISI total across the sample (Table 2, Model 1: b = 1.54 per class, 95% CI 0.92 to 2.16, *p* < 0.001), consistent with the index study [6]. This association was attenuated but remained after adjustment for benzodiazepine prescription (Model 2: b = 1.00, 95% CI 0.26 to 1.74, *p* = 0.009), with benzodiazepine prescription itself independently associated with a higher ISI (b = 1.87, *p* = 0.011).

### 3.3. Moderation, Sensitivity and Secondary Analyses

The prespecified medication complexity × mood interaction was not statistically significant (Table 2, Model 3, and Table 3: b = −1.02, 95% CI −2.25 to 0.21, *p* = 0.103) and explained negligible additional variance (ΔR^2^ = 0.005). The medication complexity slope was positive in both groups (non-mood 2.03, 95% CI 1.18 to 2.89; mood 1.01, 95% CI 0.12 to 1.90). The interaction remained non-significant after adding benzodiazepine prescription (b = −0.95, *p* = 0.127) or psychiatric comorbidity (b = −0.97, *p* = 0.123) and with HC3 robust standard errors (*p* = 0.116). In the ordinal model, the interaction was nominally significant but in the direction opposite to that hypothesised (coefficient = −0.34, 95% CI −0.68 to −0.01, *p* = 0.044); the proportional-odds assumption was not rejected (Brant test χ^2^ = 18.3, df = 12, *p* = 0.107, with *p* ≥ 0.91 for the focal terms). Given the non-significant primary test and the different outcome metric, this nominal, direction-discordant result is treated as an exploratory signal rather than as evidence of moderation, and the full ordinal model is reported in the Supplementary Material (Table S2).

Addressing H2 directly, the medication complexity × sex interaction was non-significant (b = 0.28, 95% CI −0.90 to 1.46, *p* = 0.64), indicating no evidence that sex modifies the medication complexity–insomnia association. Addressing H3, the joint model testing whether the moderation differed between the nocturnal and impact/distress domains yielded a non-significant three-way medication complexity × mood × domain interaction (b = −0.02, 95% CI −0.13 to 0.08, *p* = 0.67); on a common standardised scale, the medication complexity association was of similar magnitude in the two domains (β = 0.23 for impact/distress and β = 0.19 for nocturnal). Neither secondary test survived the Benjamini–Hochberg correction (adjusted q = 0.67 for both the medication complexity × sex interaction and the three-way interaction), and no influential observations were identified (maximum Cook’s distance = 0.021). Interaction estimates across models are summarised in Table 3.

### 3.4. Exploratory Diagnosis-Specific Analyses

As an exploratory, hypothesis-generating analysis, we estimated the medication complexity slope within each individual diagnosis (Supplementary Table S1). The point estimates were positive in every diagnostic group except adjustment disorder, and several were individually significant, but per-diagnosis samples were small and confidence intervals wide, so these estimates cannot establish that the association is uniform across diagnoses. Within the mood group, the slope did not differ between depressive and bipolar disorders (interaction *p* = 0.61).

## 4. Discussion

In this preregistered secondary analysis, the prespecified hypothesis of a stronger medication complexity–insomnia association in patients with mood disorders was not supported: the medication complexity × mood interaction was small, non-significant and, if anything, in the opposite direction. The medication complexity × sex interaction was also non-significant (notwithstanding documented sex differences in insomnia epidemiology [11]), as was the three-way test of whether moderation differed between insomnia domains. These results indicate that the hypothesised stronger association was not detected. They do not establish equivalence: because no equivalence margin was prespecified, the data remain compatible with a difference smaller than the study could detect, and the wide diagnosis-specific estimates reinforce that uniformity across the individual diagnoses cannot be claimed.

Medication complexity was associated with insomnia severity across the sample, and this association attenuated but persisted after benzodiazepine adjustment. This pattern is difficult to interpret causally. Because several counted classes, including benzodiazepines and Z-drugs, may be prescribed specifically in response to insomnia, medication complexity and the ISI are partly coupled by indication, and benzodiazepine adjustment can only partly address this because benzodiazepines are themselves part of the count. The dataset lacked information on dose, timing, indication, duration and actual use, so the association may reflect treatment complexity, prescribing in response to insomnia, or unmeasured illness severity; the present design cannot distinguish these [12,13]. The independent association of benzodiazepine prescription with higher severity is consistent with, but does not resolve, this ambiguity [14].

The nominally significant ordinal interaction, in the direction opposite to the hypothesis, is best read as a minor exploratory signal: it addresses a categorical estimand and diverges from the confirmatory continuous test. We report it in full rather than dismiss it, but it does not change the primary conclusion.

These findings do not evaluate medication complexity as a screening or predictive instrument, and we make no claim that it is a clinically useful marker; establishing that would require a prospective study with an explicit predictive framework. Likewise, although routine insomnia assessment across the outpatient caseload is reasonable on the basis of the broader literature, in which insomnia warrants treatment in its own right [15,16,17] and improving sleep can reduce psychopathology [18,19], this recommendation is supported by that literature rather than established by the present moderation analysis. Insomnia has also been linked to continued engagement with outpatient services [20], and circadian dysregulation may contribute to sleep complaints, particularly in mood disorders [21].

This study has several limitations. It is cross-sectional, so the direction of the medication–insomnia relationship cannot be established, and confounding by indication is likely. Medication was coded at the class level only; standardised measures of symptom severity and illness duration were unavailable, leaving residual confounding possible; and the sample was drawn from a single centre. The binary moderator combines heterogeneous conditions (depressive with bipolar disorders, and a mixed non-mood group), which could obscure diagnosis-specific effects, although the grouping was prespecified to preserve power; adequately powered, diagnosis-specific analyses would be required to characterise any such effects. The study was powered only for a small or larger interaction and could not exclude a very small one, and, as a secondary analysis of a previously reported cohort [6], its incremental contribution is a preregistered, confirmatory moderation test on the continuous outcome (the medication complexity × mood interaction), together with non-preregistered sex and domain analyses added during revision, all of which failed to detect the hypothesised effect. Future work should test these questions prospectively, in adequately powered, multicentre and diagnosis-specific designs that capture medication dose, indication and duration alongside standardised severity measures; the present preregistered null is most useful for informing the design and powering of such studies rather than for direct clinical recommendations.

## 5. Conclusions

In community psychiatric outpatients, we did not detect the hypothesised stronger association between medication complexity and insomnia severity in patients with mood disorders, nor any modification by sex or across insomnia domains; the analysis cannot, however, establish that the association is equivalent across groups. Benzodiazepine prescription was independently associated with insomnia severity. Given the cross-sectional design and probable confounding by indication, these preregistered null findings are best used to constrain over-interpretation of subgroup patterns rather than to support diagnosis-specific or screening claims.

## Figures and Tables

**Table 1:** Characteristics of the analytic sample (*N* = 502). Full-cohort descriptive statistics are reported in the index study [6].

Characteristic	Value
Analytic sample, *n*	502
**Continuous variables, mean ± SD**	
Age, years	45.2 ± 16.6
Education, years	12.4 ± 3.6
Medication complexity, classes	1.85 ± 1.01
ISI total score	12.2 ± 7.0
**Categorical variables, n (%)**	
Female sex	261 (52)
Benzodiazepine prescription	261 (52)
Mood disorders (depressive or bipolar)	245 (49)
Non-mood disorders	257 (51)

**Table 2:** Association between medication complexity and the ISI total score (N = 502).

Model/Term	b (95% CI)	*p*
Model 0—unadjusted †		
Medication complexity	1.66 (1.07 to 2.25)	<0.001
Model 1—main effect (adjusted) †		
Medication complexity	1.54 (0.92 to 2.16)	<0.001
Model 2—+ benzodiazepine †		
Medication complexity	1.00 (0.26 to 1.74)	0.009
Benzodiazepine prescription	1.87 (0.44 to 3.30)	0.011
Model 3—moderation (medication complexity × mood) †		
Interaction (medication complexity × mood)	−1.02 (−2.25 to 0.21)	0.103
Slope, non-mood disorders	2.03 (1.18 to 2.89)	<0.001
Slope, mood disorders	1.01 (0.12 to 1.90)	0.026
Mood disorder (main effect)	0.62 (−0.68 to 1.92)	0.349
Age (per year)	0.05 (0.02 to 0.09)	0.007
Female sex	0.91 (−0.28 to 2.10)	0.134
Education (per year)	0.05 (−0.12 to 0.21)	0.590

† Model 0 is unadjusted; Models 1 to 3 are adjusted for mood disorder status, age, sex and education. In Model 3 (moderation), with mood coded 0/1 and medication complexity centred, the “Slope, non-mood disorders” is the medication complexity coefficient and the mood slope is that term plus the interaction. Full-model R^2^ = 0.088.

**Table 3:** Medication complexity interaction estimates across sensitivity and secondary models.

Model	Interaction Estimate (95% CI)	*p*
Medication complexity × mood (primary)	−1.02 (−2.25 to 0.21)	0.103
+ benzodiazepine	−0.95 (−2.18 to 0.27)	0.127
HC3 robust SE	−1.02 (−2.30 to 0.25)	0.116
+ psychiatric comorbidity	−0.97 (−2.20 to 0.26)	0.123
Ordinal (severity categories) ‡	−0.34 (−0.68 to −0.01)	0.044
Medication complexity × sex	0.28 (−0.90 to 1.46)	0.642
Medication complexity × mood × ISI domain	−0.02 (−0.13 to 0.08)	0.672

‡ Estimates are unstandardised regression coefficients (ISI points per class) with 95% CIs, except the ordinal row, which is a log-odds coefficient, and the domain row, which is on the standardised domain scale.

## Data Availability

Anonymised data may be available from the corresponding author on reasonable request, subject to institutional approvals.

## Supplementary Materials

The following supporting information can be downloaded at https://www.mdpi.com/article/10.3390/clinpract16100189/s1, File S1: STROBE statement checklist for cross-sectional studies; Figure S1: Simple slopes; Table S1: Exploratory diagnosis-specific medication complexity slopes; Table S2: Full ordinal (proportional-odds) model.
