# Supplementary material for: Medication Complexity and Insomnia Severity in Psychiatric Outpatients: A Moderation Analysis Across Mood and Non-Mood Disorders
Source: Clin Pract. 2026 Oct 12;16(10):189. doi: 10.3390/clinpract16100189 (PMC13703539; doi:10.3390/clinpract16100189)
Supplement: Supplementary file 1 [file clinpract-16-00189-s001.zip › STROBE_checklist_2.pdf]

STROBE statement: checklist for cross-sectional studies

Manuscript: "Medication complexity and insomnia severity in psychiatric outpatients: a moderation analysis across mood and non-mood disorders". Section numbers refer to the manuscript.

Item	Topic	STROBE recommendation (cross-sectional studies)	Where addressed
**Title and abstract**			
1a	Title and abstract	State the study design with a commonly used term in the title or the abstract	Title; Abstract (Methods: cross-sectional secondary analysis)
1b	Abstract	Provide an informative and balanced summary of what was done and what was found	Abstract (Background, Methods, Results, Conclusions)
**Introduction**			
2	Background and rationale	Explain the scientific background and rationale	Introduction, paragraphs 1 to 3
3	Objectives	State specific objectives, including prespecified hypotheses	Introduction, paragraph 4 (H1, H2, H3); OSF registration z6vu8
**Methods**			
4	Study design	Present key elements of study design early	Section 2.1
5	Setting	Describe the setting, locations and relevant dates	Section 2.1 (CMHC Bra, ASL Cuneo 2; 1 September to 30 November 2025)
6	Participants	Give eligibility criteria and the sources and methods of selection	Section 2.1 (adults 18+; consecutive; one observation per patient; no further selection; eating disorders group excluded, prespecified)
7	Variables	Define outcomes, exposures, predictors, confounders and effect modifiers	Section 2.2 (ISI total and domains; medication complexity, class-level; mood status and sex as moderators; covariates; benzodiazepine)
8	Data sources and measurement	Give sources and methods of assessment for each variable	Section 2.2 (ISI, validated Italian version; SCID-5; class-level medication coding without dose/indication)
9	Bias	Describe efforts to address potential sources of bias	Sections 2.3, 3.3 (sensitivity analyses; FDR); Discussion (confounding by indication; reverse causation)
10	Study size	Explain how the study size was arrived at	Section 2.1 (analytic sample 502); Section 2.3 (fixed sample; minimum detectable effect $f^2 \approx 0.016$)
11	Quantitative variables	Explain how quantitative variables were handled and groupings chosen	Sections 2.2, 2.3 (grand-mean centring; mood vs non-mood grouping prespecified; standardised ISI domains)
12a	Statistical methods	Describe all statistical methods, including control for confounding	Section 2.3 (linear regression with interaction; covariates; IBM SPSS v22)
12b	Interactions and subgroups	Describe methods to examine subgroups and interactions	Section 2.3 (medication complexity × mood; medication complexity × sex; joint medication complexity × mood × domain model; exploratory diagnosis-specific slopes)
12c	Missing data	Explain how missing data were addressed	Sections 2.1, 3.1 (complete data; no imputation required)
12d	Sampling strategy	State how the sampling strategy was taken into account	Section 2.1 (consecutive enrolment within a fixed window)

12e	Sensitivity analyses	Describe any sensitivity analyses	Sections 2.3, 3.3 (benzodiazepine; psychiatric comorbidity; HC3 robust SE; ordinal model; Benjamini–Hochberg FDR)
**Results**			
13	Participants	Report numbers at each stage of the study	Section 3.1; Table 1 (N = 502; eating disorders group n = 4 excluded)
14	Descriptive data	Give participant characteristics and information on exposures	Section 3.1; Table 1 (grouped by scale; full cohort descriptives in the index study)
15	Outcome data	Report numbers of outcome events or summary measures	Section 3.1; Table 1 (ISI total, by mood and non-mood group)
16	Main results	Give unadjusted and confounder-adjusted estimates and their precision (95% CIs)	Section 3.2; Table 2 (unadjusted medication complexity estimate 1.66; adjusted main effect 1.54; benzodiazepine-adjusted 1.00; moderation model)
17	Other analyses	Report subgroup, interaction and sensitivity analyses	Sections 3.3, 3.4; Table 3 (interaction across models, incl. sex and domain); Supplementary Tables S1, S2; Figure S1
**Discussion**			
18	Key results	Summarise key results with reference to objectives	Discussion, paragraph 1
19	Limitations	Discuss limitations and sources of potential bias or imprecision	Discussion, final paragraph
20	Interpretation	Give a cautious overall interpretation	Discussion, paragraphs 1 to 4; Section 5 (Conclusions)
21	Generalisability	Discuss the generalisability of the results	Discussion (single centre; community outpatient setting)
**Other information**			
22	Funding	Give the source of funding and the role of funders	Declarations (Funding)

Note. Recommendations are summarised. The full STROBE statement is available at www.strobe-statement.org.
