# Supplementary material for: Medication Complexity and Insomnia Severity in Psychiatric Outpatients: A Moderation Analysis Across Mood and Non-Mood Disorders
Source: Clin Pract. 2026 Oct 12;16(10):189. doi: 10.3390/clinpract16100189 (PMC13703539; doi:10.3390/clinpract16100189)
Supplement: Supplementary file 1 [file clinpract-16-00189-s001.zip › Supplementary_Material_2.pdf]

Supplementary Material

Medication complexity and insomnia severity in psychiatric outpatients: a moderation analysis across mood and non-mood disorders

Table S1. Exploratory diagnosis-specific medication complexity slopes

Slope of medication complexity (ISI points per additional class) on the ISI total score, estimated separately within each diagnostic group, each adjusted for age, sex and education.

Table S1. Medication complexity slope on the ISI total within each diagnostic group (N = 502).

Diagnostic group	n	Slope, b (95% CI)	p
Adjustment disorder	29	−0.41 (−4.58 to 3.75)	0.839
Anxiety disorders	84	1.35 (−0.45 to 3.15)	0.139
Depressive disorders	120	1.07 (−0.50 to 2.65)	0.181
Bipolar disorders	125	1.40 (0.15 to 2.64)	0.028
Psychotic disorders	52	2.14 (−0.12 to 4.39)	0.063
ADHD	19	2.19 (−2.21 to 6.60)	0.303
Obsessive–compulsive disorder	31	3.36 (0.59 to 6.14)	0.019
Personality disorders	42	2.61 (1.22 to 4.00)	0.001

Per-diagnosis samples are small and confidence intervals wide; these exploratory estimates cannot establish that the association is uniform across diagnoses. Within the mood group, the slope did not differ between depressive and bipolar disorders (medication complexity × subgroup interaction b = 0.51, 95% CI −1.45 to 2.47, p = 0.61).

Table S2. Full ordinal (proportional-odds) model

Ordinal logistic regression of the four ISI severity categories on the medication complexity × mood interaction, adjusted for age, sex and education. Coefficients are on the log-odds scale.

Table S2. Ordinal model of ISI severity categories (N = 502).

Term	Coefficient (95% CI)	p
Medication complexity	0.54 (0.31 to 0.78)	<0.001
Mood disorder	0.19 (−0.17 to 0.54)	0.300
Medication complexity × mood	−0.34 (−0.68 to −0.01)	0.044
Age	0.013 (0.003 to 0.023)	0.014
Female sex	0.23 (−0.10 to 0.55)	0.172
Education	0.008 (−0.036 to 0.052)	0.736

Three threshold (cut-point) parameters were estimated (model log-likelihood = −643.6; N = 502). The proportional-odds assumption was assessed with a Brant test and was not rejected overall (χ^2 = 18.3, df = 12, p = 0.107); it held for the focal terms (medication complexity, mood and their interaction, all p ≥ 0.91), with non-proportionality only for age (p = 0.005), a covariate. The interaction is nominally significant but in the direction opposite to the hypothesis; it is interpreted as an exploratory signal, not as evidence of moderation.

Figure S1. Simple slopes

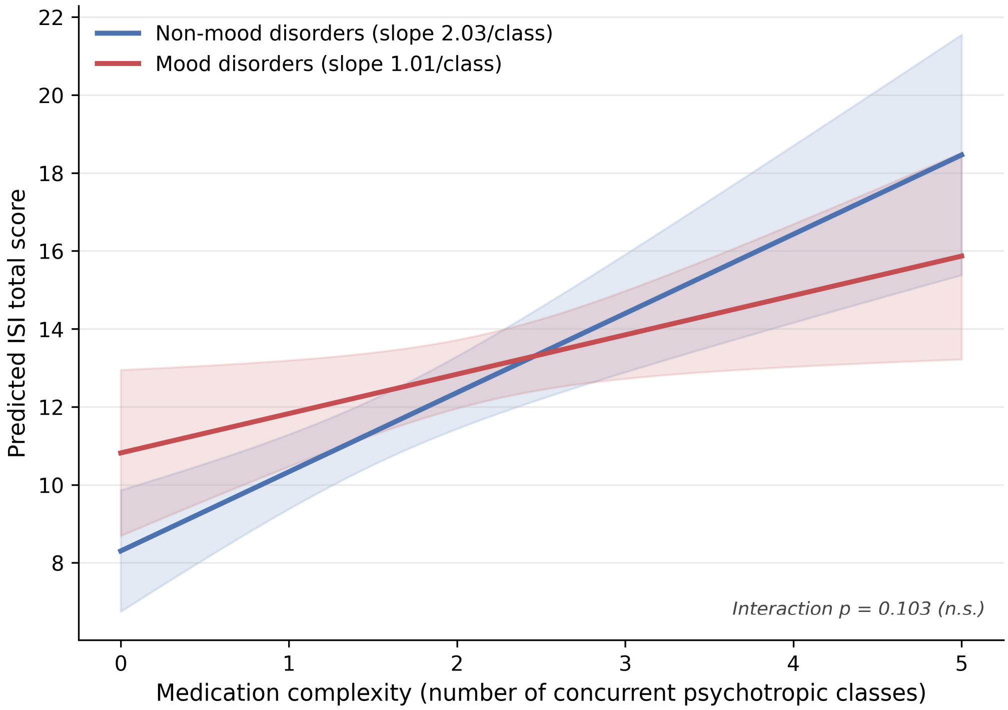

Figure S1. Predicted ISI total score as a function of medication complexity, for patients with mood and non-mood disorders separately, from the primary linear model (covariates held at sample means). Shaded bands are 95% confidence intervals. The medication complexity × mood interaction was not significant ($p = 0.103$).
